# Modeling of RC-IGCT-based superconducting hybrid DC circuit breaker with TP KEMA arc model and hybrid SFCL

**DOI:** 10.1038/s41598-026-50078-0

**Published:** 2026-04-24

**Authors:** Muhammad Junaid, Xiaoting Wang, Dongsheng Yu, Yiwei Peng

**Affiliations:** School of Electrical Engineering, China University of Mining and Technology, Xuzhou, 221116 China

**Keywords:** Energy science and technology, Engineering

## Abstract

Achieving rapid fault clearance in medium-voltage direct current (MVDC) grids requires DC circuit breakers (DCCBs) with high current-interrupting capability and low cost. Our team previously proposed a hybrid DC circuit breaker combining Asymmetric IGCT (A-IGCT) and resistive superconducting fault current limiters (R-SFCL). To further optimize this design, this paper introduces an improved superconducting hybrid DC circuit breaker (SDCCB) based on reverse-conducting integrated gate commutated thyristors (RC-IGCT). Key enhancements include integrating a hybrid superconducting fault current limiter (H-SFCL) and employing the TP KEMA arc model. This combination strengthens current limiting capability and more accurately simulates arc dynamics during commutation, achieving superior breaking capacity while maintaining low cost. Simulation evaluations assess fault current breaking performance and the influence of circuit parameters. Comparative analysis with A-IGCT and IGBT-based power semiconductor devices demonstrates that RC-IGCT is the most suitable solid-state switch for this topology in VSC-MVDC systems. Results confirm the proposed circuit breaker can interrupt 21.9 kA fault currents within 3.5 ms.

## Introduction

As a key direction in the evolution of future power systems, DC grids demonstrate broad application prospects across multiple sectors due to their superior transmission capacity, flexible controllability, and high supply reliability^1–3^. Among these, MVDC technology is progressively playing a pivotal role in distribution networks, garnering particular attention in railway systems, marine power applications, offshore renewable energy collection, and distribution grids^4,5^. DC grids must ensure reliable energy transmission, necessitating rapid fault clearance to minimize system disruption. Consequently, DC circuit breakers with high-performance interrupting capabilities and low operational costs are essential.

Currently, DC circuit breakers can be primarily categorized into three technical approaches: mechanical, solid-state, and hybrid^6–8^. Among these, hybrid DC circuit breakers combine the rapid breaking capability of solid-state breakers with the low conduction losses of mechanical breakers, making them the mainstream direction in current technological development^9^. However, their performance and cost remain constrained by the selection of power electronic devices and the choice of topological structures. To overcome this bottleneck, researchers have introduced superconducting technology^10^.

However, existing superconducting hybrid solutions involve trade-offs between cost and performance: U. Amir Khan et al.^11^ proposed coordinating R-SFCLs with conventional hybrid circuit breakers to restrict main branch currents, achieving 11 kA fault current interruption within 6 milliseconds. However, in this topology, both the line converter switch and the main circuit breaker employ an IGBT mirror-pair configuration for current conversion and fault current interruption, significantly increasing the cost of power electronic components and the hybrid circuit breaker^12,13^. Zhuang Weibin et al.^14^ proposed a novel DC circuit breaker topology based on reverse current injection and IGCT technology, capable of interrupting 20 kA fault currents. However, its interrupting capability and speed still have room for improvement. Yang Xu et al.^15^ proposed a superconducting DC circuit breaker based on controllable current conversion, capable of interrupting a fault current of 24.2 kA within 4.11 ms. This circuit breaker further optimizes costs by eliminating the pre-charging circuit through an H-bridge structure, albeit at the expense of interrupting speed. Our team proposed an A-IGCT based superconducting hybrid DC circuit breaker integrating an R-SFCL with a reverse-current-injection circuit breaker, capable of interrupting 17.6 kA fault currents within 3.8 ms^16,17^.

Despite its remarkable performance, the employed R-SFCL inherently faces hot-spot issues in superconducting bands. In non-superconducting state, the R-SFCL enters a high-resistance state. Prolonged fault current exposure leads to overheating and damage due to accumulated Joule heating, fundamentally limiting the sustainability of high-current interruptions^18^. These studies indicate that maintaining or enhancing interrupting speed while utilizing low-cost components remains a critical challenge in current research.

To effectively address the aforementioned issues, this paper proposes and analyzes a novel superconducting hybrid DC circuit breaker based on RC-IGCT technology. This solution aims to leverage the low-cost advantage of RC-IGCT while achieving reliable high-current interruption at enhanced breaking speeds through the coordinated optimization of the main current, transfer, active current injection, and energy absorption branches. This approach delivers an optimal balance between cost and performance.

The structure of this paper is as follows: Sect.  2 describes the A-IGCT-based superconducting hybrid DC circuit breaker previously proposed by our team, focusing on its A-IGCT functional simulation model, R-SFCL, and Mayr arc model. Section  3 first introduces the selection criteria for solid-state switches, the turn-on and turn-off transient simulation circuit for RC-IGCT, and the topology and operating mechanism of the superconducting current limiter. Subsequently, it details the proposed circuit breaker topology and operating principle. Section  4 applies the circuit breaker to a VSC-MVDC system, simulates and analyzes its interrupting performance, compares the RC-IGCT-based solution with those based on A-IGCT and IGBT, and evaluates the proposed circuit breaker’s interrupting capability. It also analyzes the current-limiting effects of different SFCLs and the impact of circuit parameters on performance. Section  5 discusses costs from three perspectives: the current limiting module, the MOV module, and the main power electronic switch. Finally, Sect.  6 summarizes the research content of this paper.

## Previous investigations on superconducting hybrid DC circuit breaker based on IGCT

Our team previously proposed a superconducting hybrid DC circuit breaker based on A-IGCT, utilizing IGCT as the solid-state switch and employing a resistive superconducting fault current limiter to restrict fault currents. In MATLAB/Simulink simulations, the Mayr arc model was used to simulate the Ultra-fast disconnect switch (UDS).

**Fig. 1:**
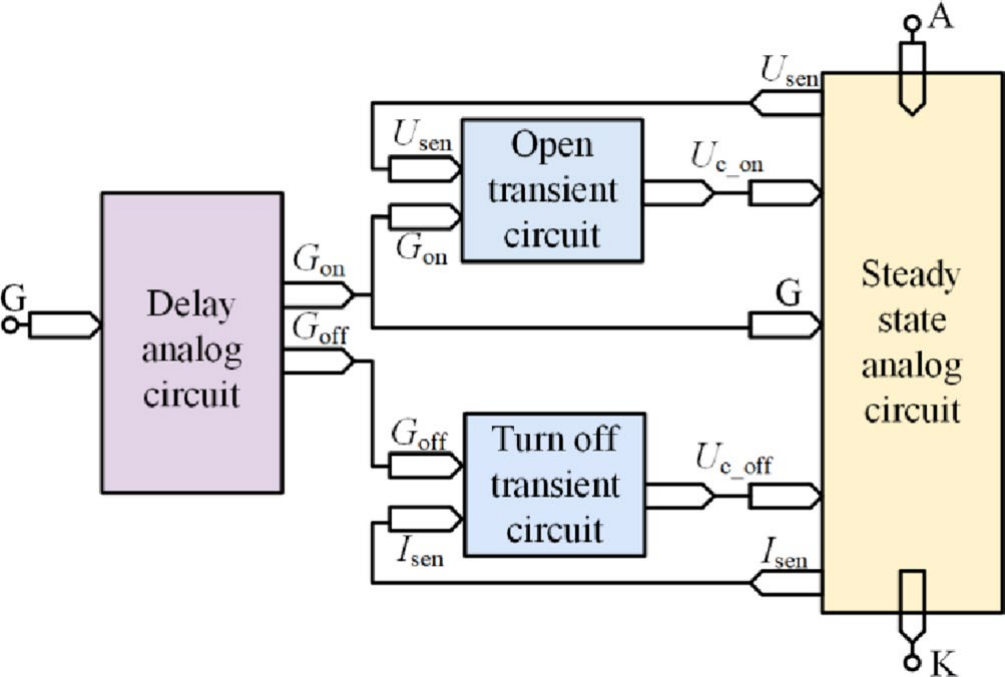
A-IGCT functional simulation model.

According to existing literature, IGCT device models are categorized into three types: ideal switches, functional simulation models, and compact models based on physical processes^19–23^. Among these, functional simulation models utilize circuit elements to simulate the static and dynamic characteristics of the device, making them suitable for power circuit topology and parameter design. The functional simulation models for A-IGCT are shown in Fig. 1. It includes: a delay simulation circuit, a turn-on transient circuit, a turn-off transient circuit, and a steady-state simulation circuit.

**Fig. 2:**
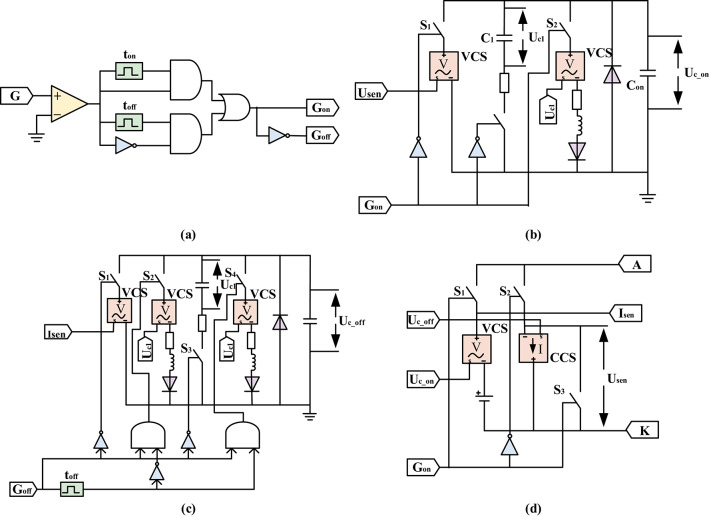
A-IGCT functional simulation model circuit modules (**a**) A-IGCT trigger signal delay module (**b**) A-IGCT turn-on transient module (**c**) A-IGCT turn-off transient module **(d**) A-IGCT steady-state simulation circuit.

Figure 2 (a) shows the trigger signal delay module circuit. In this circuit, G represents the gate trigger signal, G_on_ is the enable signal for the turn-on voltage source branch, and G_off_ is the enable signal for the turn-off current source branch. All enable signals activate at a high level. The turn-on delay time ton and turn-off delay time toff are approximately several microseconds. Figure 2 (b) shows the turn-on transient circuit. It consists of a controllable ideal switch and a controlled voltage source module. The ideal bidirectional controllable switch is used to switch the turn-on branch. The branch containing the Gon-controlled ideal switch is the turn-on branch. U_c_on_ represents the output of the turn-on transient circuit. Figure 2 (c) shows the turn-off transient circuit. The branch containing the Goff-controlled ideal switch is the turn-off branch. U_c_off_ is the output of the turn-off transient circuit. Figure 2 (d) illustrates the steady-state analog circuit. A denotes the anode, K denotes the cathode; I_sen_ and U_sen_ represent the current flowing through the controlled voltage source and the voltage across the controlled current source in the steady-state analog circuit, respectively, used to complement the transient circuit simulation.

In the proposed circuit breaker, the resistive superconducting fault current limiter can transition to its maximum current-limiting resistance value of 9 Ω within 0.2ms. It is capable of limiting a 17.6 kA fault current to within the range of 2.1 kA.

In the circuit breakers under study, arcing occurs during the UDS current interruption. Based on this, the Mayr arc model was employed to simulate the UDS. The equations of the Mayr model are:

1 $$\frac{{{\mathrm{d}}g}}{{dt}} = \frac{g}{\tau }\left( {\frac{{ui}}{P} - 1} \right)$$

### A novel superconducting hybrid DC circuit breaker

#### Solid-state switching semiconductor device RC-IGCT

**Fig. 3:**
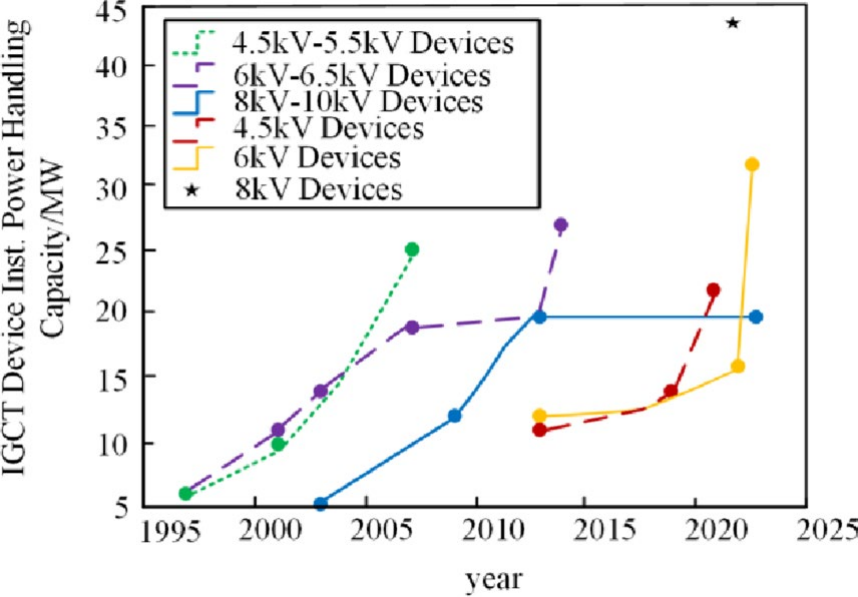
Evolution of instantaneous power handling capability in IGCT devices.

Among various power semiconductor devices, the IGCT plays an indispensable role in medium- and high-voltage DC circuit breakers due to its inherent advantages of high voltage withstand capability, large current carrying capacity, high reliability, and low cost^24^. Figure 3 illustrates the evolution of transient power handling capability in IGCT devices over the past two decades^25^. Based on voltage ratings, IGCTs are primarily categorized into three tiers: 4.5–5.5 kV, 6-6.5 kV, and 8–10 kV.

**Fig. 4:**
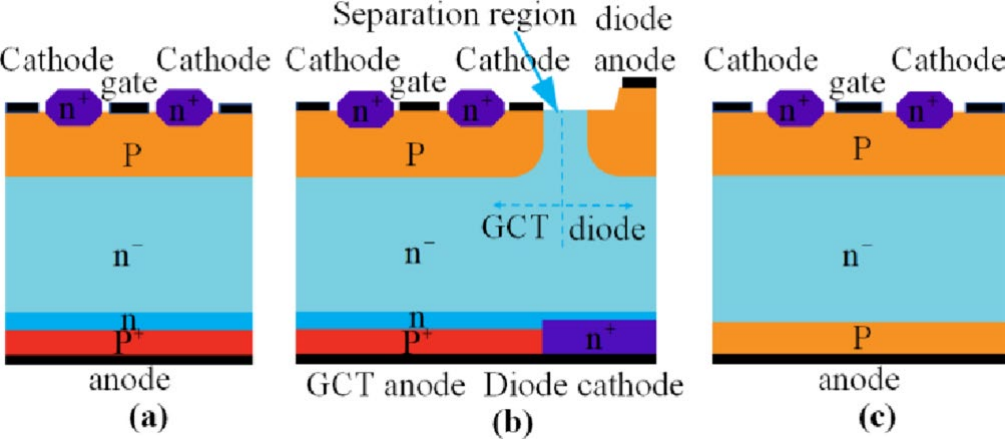
IGCT chip structure comparison: (**a**) A-IGCT; (**b**) RC-IGCT; (**c**) RB-IGCT.

IGCT can be categorized into A-IGCT, RC-IGCT, and reverse-blocking IGCT (RB-IGCT) based on their functional characteristics, as illustrated in Fig. 4^26^. The schematic structure of A-IGCT is shown in Fig. 4(a). This semiconductor cannot block reverse voltage nor provide reverse conduction. For A-IGCTs, the maximum repetitive reverse blocking voltage is far lower than the forward blocking voltage^27^. The chip of RC-IGCT consists of an integrated structure formed by the GCT section and a diode section connected in reverse parallel, as shown in Fig. 4(b). Its GCT section is fundamentally identical to the asymmetric GCT structure, while the integrated reverse-parallel diode facilitates reverse current flow. The structural schematic of the RB-IGCT is shown in Fig. 4(c). This semiconductor possesses reverse voltage blocking capability, with its reverse blocking voltage rating being comparable to its forward blocking voltage rating.

Considering that the operating principle and application scenarios of RC-IGCT are similar to those of A-IGCT, while integrating both GCT and reverse-parallel diodes onto the same chip compared to the external reverse-parallel diodes of A-IGCT, RC-IGCT offers higher power density and simplifies external circuitry and installation structures. Therefore, this paper focuses on RC-IGCT and applies it to the proposed DC circuit breaker topology.

**Fig. 5:**
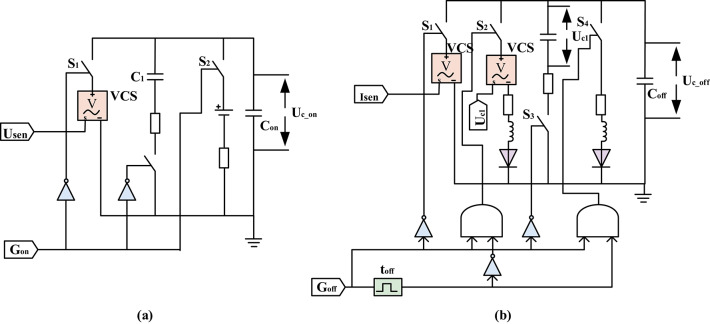
RC-IGCT functional simulation model turn-on and turn-off modules (**a**) RC-IGCT turn-on transient simulation circuit (**b**) RC-IGCT turn-off transient simulation circuit.

Figure 5 (a) shows the equivalent voltage source simulation circuit for the RC-IGCT turn-on process in MATLAB/Simulink. The turn-on process treats the RC-IGCT as a variable voltage source with a voltage that decreases approximately linearly, simulated using a first-order RC circuit. Figure 5 (b) shows the simulation circuit for the RC-IGCT turn-off process. The turn-off process treats the RC-IGCT as a variable current source, modeled using a two-stage second-order RLC circuit. The duration of the first stage is determined by the fall time, while the duration of the second stage is determined by the tail current time.

#### TP KEMA arc model for the UDS

Arc is a key physical process affecting the commutation performance of circuit breakers. In black-box models, mathematical equations are commonly used to describe its external characteristics. Our team previously employed the Mayr model to simulate the UDS arc, which performs well in the low-current region but becomes excessively linear near the current zero-crossing point, making it difficult to accurately capture the sharp increase in arc resistance and the nonlinear characteristics of the commutation process. To address this, researchers have proposed improved models such as the Habedank and Schwarz models. In this paper, the TP KEMA arc model is adopted, which achieves structural simplification while retaining the high accuracy of the KEMA model, providing a more reliable simulation foundation for the commutation analysis of circuit breakers. The equations for the TP KEMA model are given in^28^:

2 $$\left\{ {\begin{array}{*{20}c} {\frac{{dg_{1} }}{{dt}} = \frac{1}{{10^{6} TP}}g_{1}^{{1.4}} u_{1}^{2} - \frac{1}{{25T}}g_{1} } \\ {\frac{{dg_{2} }}{{dt}} = \frac{1}{{10^{3} TP}}g_{2}^{{1.9}} u_{2}^{2} - \frac{1}{{5T}}g_{2} } \\ {\frac{{dg_{3} }}{{dt}} = \frac{1}{{TP}}g_{3}^{2} u_{3}^{2} - \frac{1}{T}g_{3} } \\ \end{array} } \right.$$

**Fig. 6:**
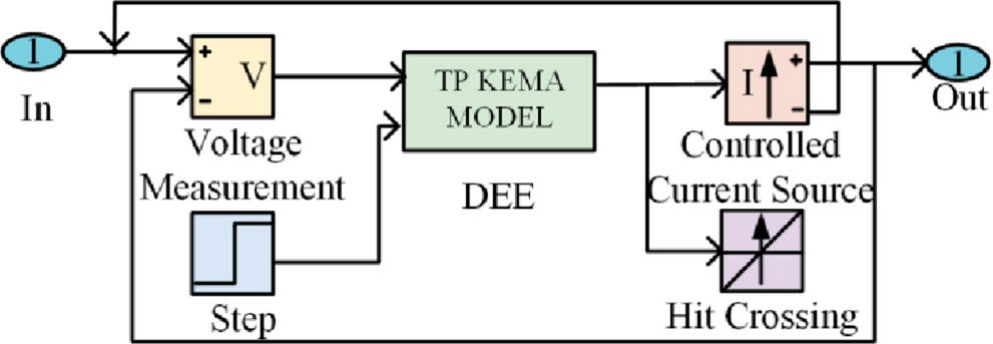
The TP KEMA arc model in MATLAB/Simulink.

Figure 6 shows the TP KEMA model in MATLAB/Simulink. The arc equation is implemented using the Differential Equation Editor (DEE), with voltage and trip time as inputs and current as the output. A controlled current source, driven by the DEE output, represents the arc current. The UDS operation signal is generated in a stepwise manner, allowing precise localization of the arc current zero-crossing by fine-tuning the step increment.

### Topology and principle of superconducting fault current limiters

**Fig. 7:**
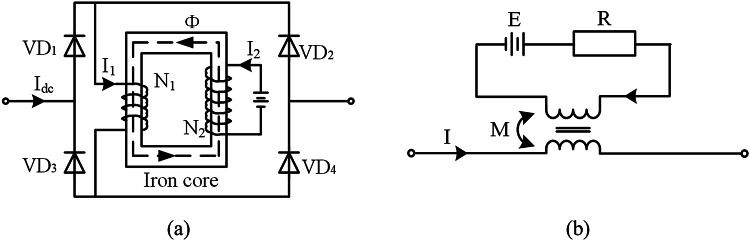
The topological structure of the SFCL (**a**) Topological Structure of SI-SFCL (**b**) Topological Structure of Novel-SFCL.

Figure 7 (a) illustrates the structure of the SI-SFCL. The overall assembly comprises a diode bridge circuit (VD1-VD4), a conventional primary-side coil, a superconducting secondary-side coil, a DC power source, and a core. The core serves as the central component of the entire device, with the SI-SFCL leveraging its nonlinear magnetic properties to influence the impedance characteristics of the current limiter. During this phase of core permeability variation, the equivalent inductance of the SI-SFCL can be expressed as^29^:

3 $$L = \frac{{N_{1}^{2} S}}{l}\left( {\mu - \frac{{N_{2} I_{2} - N_{1} I_{1} }}{{N_{1} }} \cdot \frac{{d\mu }}{{dI_{1} }}} \right)$$

The Superconducting Power Research Center at Huazhong University of Science and Technology proposed a novel topological design for a DC superconducting current limiter^30^, as shown in Fig. 7 (b). Here, E represents the DC power source, R denotes the load circuit resistance, and the DC superconducting fault current limiter consists of two superconducting coupled coils wound around a single iron core. During normal operation, the two superconducting coils carry currents of equal magnitude but opposite direction. The magnetic fluxes generated by the coils cancel each other out, resulting in zero magnetic flux in the iron core. Thus, the current limiter has no effect on the system. In the event of a short-circuit fault, the load circuit current increases, and the short-circuit current is limited by the inductance of the superconducting coils.

**Fig. 8:**
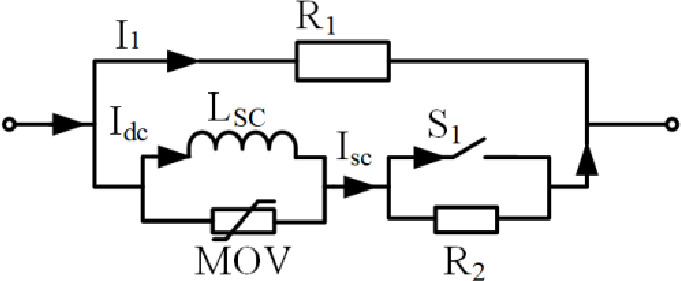
Topological Structure of H-SFCL.

The topological configuration of a hybrid superconducting current limiter^31^ is shown in Fig. 8. This current limiter consists of resistors R_1_ and R_2_, switch S_1_, and superconducting inductor Lsc. Current transfer is achieved via resistor R_1_, with initial current limiting performed by superconducting inductor Lsc. After switch S_1_ opens, resistor R_2_ is connected in series to the system for subsequent current continuation. During the early stage of current limiting, the inductive component restricts the initial peak of the short-circuit current, while in the later stage, the resistive component suppresses the steady-state short-circuit current, providing a certain level of current limiting capability.

**Fig. 9:**
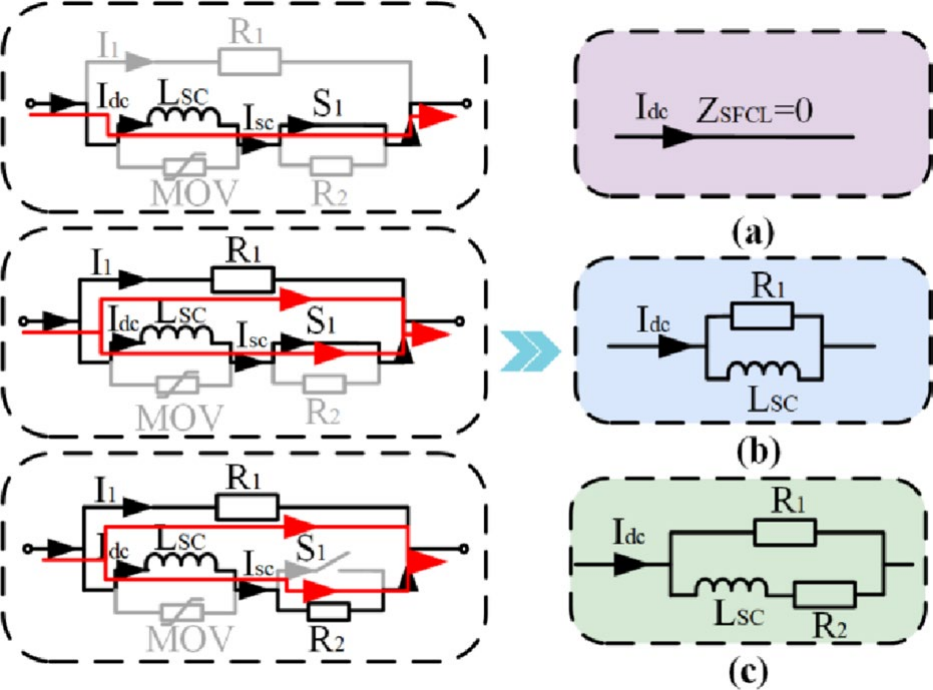
Equivalent circuit of the H-SFCL under different operational states: (**a**) Normal operation; (**b**) Initial fault current limiting; (**c**) Full current limiting.

The operational process of the H-SFCL and its corresponding equivalent circuit are illustrated in Fig. 9.

### Topology and principles of a novel RC-IGCT-based superconducting hybrid DC circuit breaker

**Fig. 10:**
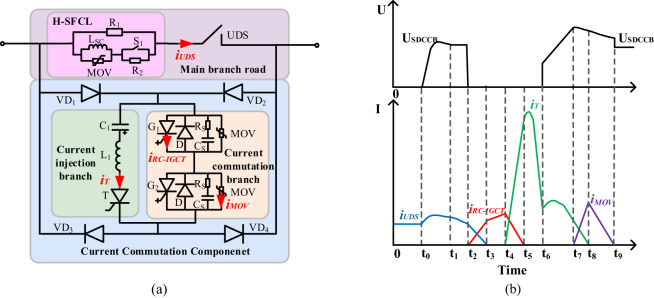
The topological structure of SDCCB and typical current commutation and voltage waveforms during interruption (**a**) Topology of a Superconducting Hybrid DC Circuit Breaker Based on RC-IGCT (**b**) Current and Voltage Waveforms of Main Components During SDCCB Current Interruption Process.

Figure 10 (a) illustrates a hybrid DC circuit breaker topology integrating RC-IGCT and superconducting technology, comprising a main current path and an energy transfer path. The main current path incorporates a series-connected current-limiting module formed by H-SFCL and an UDS. During a fault, the H-SFCL initiates operation to suppress the fault current; followed by the UDS for rapid physical isolation and current reversal. The energy transfer path primarily handles current interruption and includes: a bridge converter module (VD_1_-VD_4_) providing bidirectional current conduction capability; an active current injection branch, consisting of a series connection of a precharge capacitor, inductor, and thyristor, used to generate reversing current and establish voltage. Transfer current branch components, centered on RC-IGCT, supplemented by a freewheeling diode, RC buffer circuit, and an energy absorption branch composed of metal oxide surge suppressors (MOV).

**Fig. 11:**
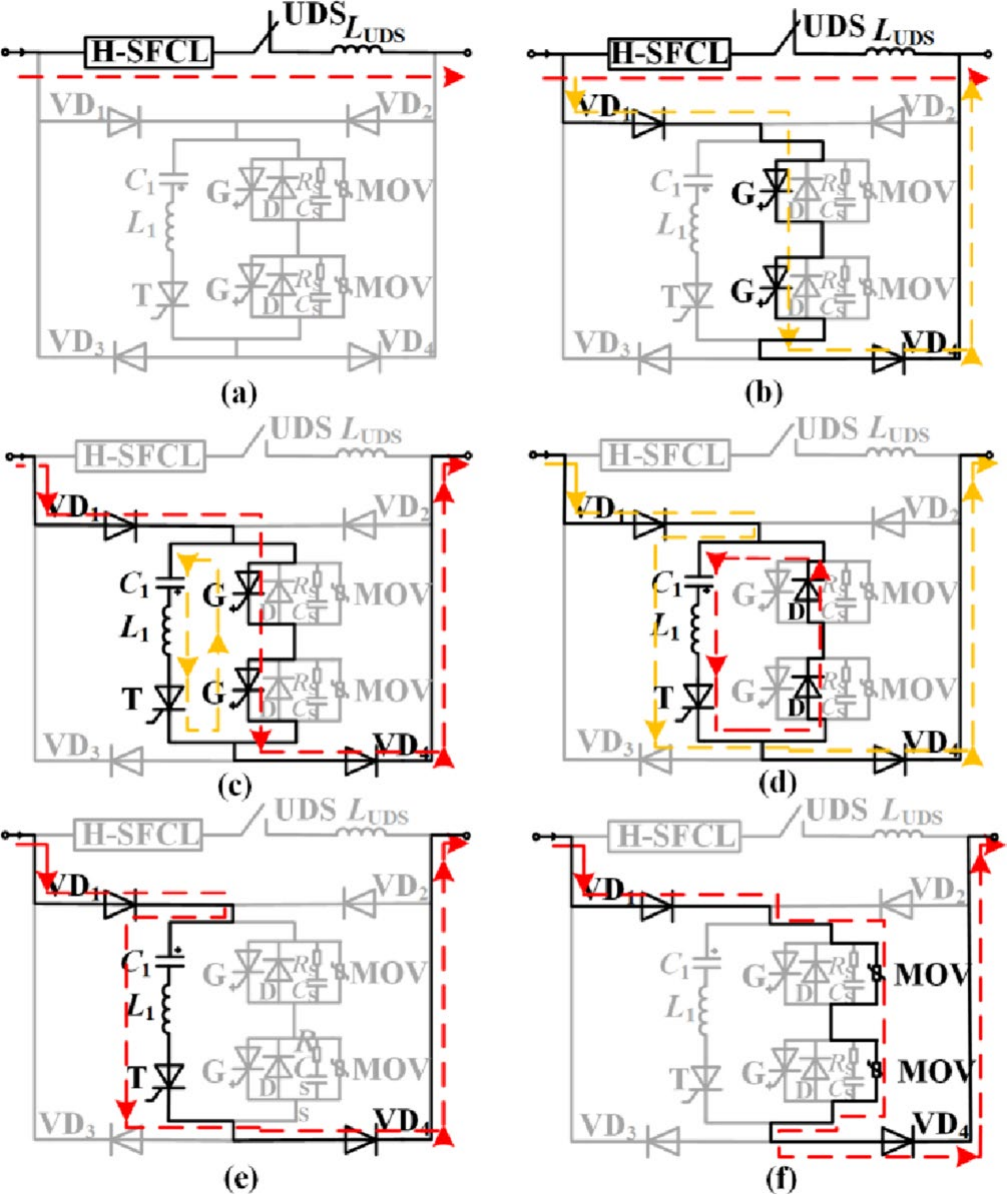
Current Variation Process During SDCCB Disconnection. (**a**) System fault occurrence (**b**) First current reversal after UDS operation (**c**) Second current reversal after capacitor C1 discharge (**d**) Diode freewheeling during second current reversal (**e**) Reverse charging of capacitor C_1_ after RC-IGCT shutdown (**f**) Energy release from system fault.

For the topology shown in Fig. 10 (a), Fig. 10 (b) illustrates the current and voltage waveforms of the main components during the SDCCB current-breaking process. Specifically, i_UDS_ represents the current in the UDS, i_RC-IGCT_ denotes the current in the RC-IGCT, i_T_ indicates the thyristor current flowing in the active current injection branch, and i_MOV_ corresponds to the current in the MOV. U_SDCCB_ denotes the voltage across the SDCCB terminals. Figure 11 presents a schematic diagram of current and voltage during the fault current interruption process. The current variation during DC circuit breaker tripping can be divided into the following six stages.

(1) Main Branch Conduction Phase (t_0_-t_2_). At time t_0_, a system fault occurs. Current flows through the main branch, with H-SFCL and UDS conducting current at a level no lower than the main branch overcurrent protection setting. During this phase, no current flows through the energy transfer path, and both ends bear the main branch conduction voltage, as shown in Fig. 11 (a).

(2) Current Transfer Stage 1 (t_2_-t_3_). Upon receiving the break command, the circuit breaker locks out the main path (at time t_2_). The UDS remains closed, initiating current transfer to the rated current transfer path. The rated current transfer path remains conductive until the main path current crosses zero (at time t_3_). This stage is illustrated in Fig. 11 (b).

(3) UDS Disconnection Phase (t_3_-t_4_). After the main branch current crosses zero, the UDS begins disconnection until sufficient clearance is established at the UDS breakers, at which point the transfer branch is locked out (at time t_4_). At this moment, capacitor C_1_ discharges into the RC-IGCT, providing a reverse voltage to cause its turn-off. This phase is illustrated in Fig. 11 (c).

(4) Current Transfer Phase 2 (t_4_-t_6_). Current begins to transfer from the RC-IGCT branch to the current injection branch containing thyristor T. When the current in the RC-IGCT drops to zero, the RC-IGCT reliably turns off at a low current level. After i_T_ reaches its maximum value, capacitor C_1_ is reverse-charged by the fault current. At this point, the voltage across the SDCCB begins to rise. This phase is illustrated in Fig. 11 (d).

(5) Energy Absorption Branch MOV Operation Phase (t_6_ ~ t_8_). After the transfer current branch is locked out, current flows through the module capacitor. When the transient voltage established in the transfer branch exceeds the MOV operating voltage, current gradually shifts from the transfer branch to the energy absorption branch MOV until the transfer branch current crosses zero (at time t_8_). This phase is illustrated in Fig. 11 (e).

(6) MOV Current Decay Phase (t_6_ ~ t_9_). After the fault current transfers to the energy-absorbing branch MOV, the transient voltage and current established by the circuit breaker gradually decay to zero. During this process, the MOV absorbs the remaining energy in the system. Following the MOV current crossing zero, the circuit breaker terminals bear the DC system voltage, as shown in Fig. 11 (f).

### Turn-off modes and characteristics analysis of the novel SDCCB

#### SDCCB fault interruption characteristics

**Fig. 12:**
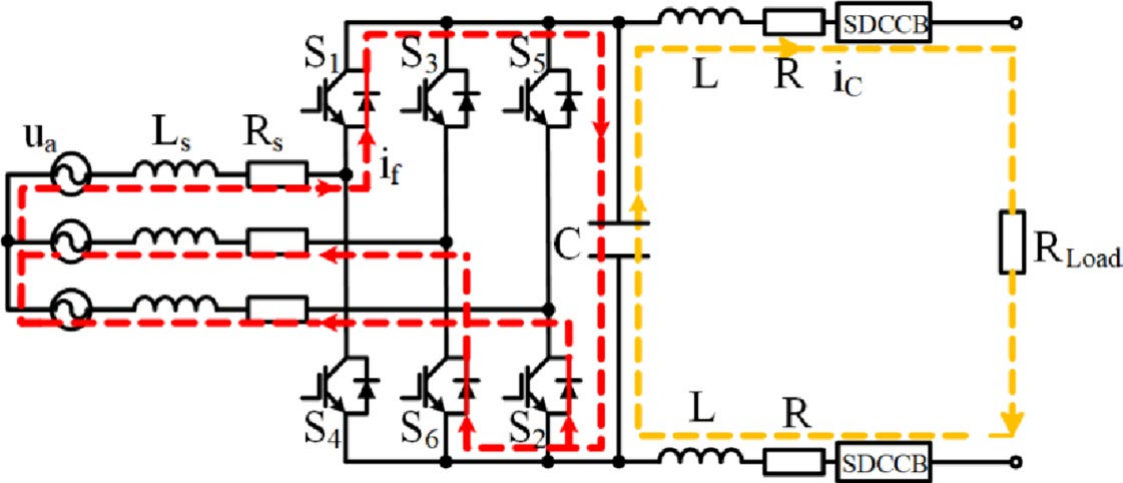
10 kV VSC-MVDC System Simulation Model.

Single-pole ground faults and bipolar short-circuit faults represent the two primary fault modes in flexible DC transmission lines. Transient and permanent single-pole ground faults, along with bipolar short-circuit faults, constitute the main causes of overcurrent in DC transmission lines. Considering the operational performance of the new SDCCB under the most severe fault current conditions, this study analyzes its interruption characteristics during bipolar short-circuit faults. This paper constructs a 10 kV VSC-MVDC system simulation model, where resistive loads represent the load side. A bipolar short-circuit fault occurs on the DC side, as shown in Fig. 12. Key parameters of medium-voltage DC systems and SDCCBs are shown in Table 1.

**Table 1:** Key parameters of medium-voltage DC systems and SDCCBs.

Parameters	Value
Rated voltage	10 kV
Rated current	1 kA
Rated power	10 MW
di/dt	13.7 kA/ms
Fault current after H-SFCL limit	4.2 kA
System inductance (L)	660 µH
R_H−SFCL_ (R_1_)	3 Ω
R_H−SFCL_ (R_2_)	8 Ω
L_H−SFCL_ ( L_SC_)	3 mH
Pre-charged capacitance (C_1_)	0.3 mF
Pre-charged voltage of capacitance (U_C1_)	5 kV
Inductance in the active current (L_1_)	4 µH
Snubber capacitance (C_S_)	10 µF
Snubber resistance (R_S_)	5 Ω
Operating voltage of MOV (U_MOV_)	6.5 kV

**Fig. 13:**
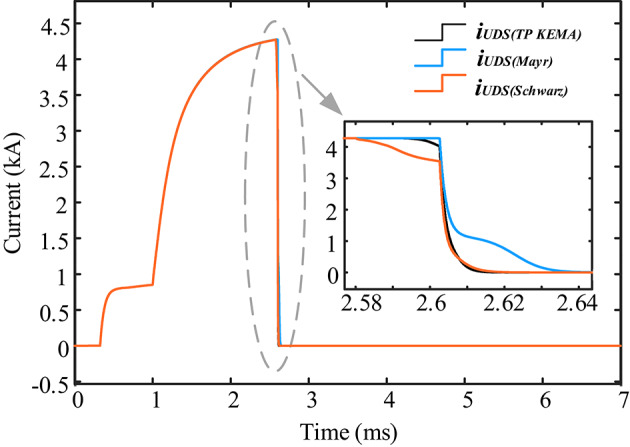
Enlarged comparison graph of the main branch current i_UDS_ near the zero-crossing point under different arc models.

Figure 13 presents an enlarged comparison graph of the main branch current i_UDS_ near the zero-crossing point under different arc models. The Mayr model exhibits overly linear behavior near the zero-crossing point, failing to capture the nonlinear characteristics of the sharp increase in arc resistance. The Schwarz model suffers from numerical oscillations when handling high-voltage, high-current commutation. In contrast, the TP KEMA model effectively captures the “squeezing” effect before current zero crossing and accurately simulates the physical process in which the rise in arc voltage facilitates current commutation to the RC-IGCT transfer branch, demonstrating greater consistency with actual operating conditions in terms of computational stability and dielectric recovery characteristics. Simulation results show that the TP KEMA model provides a more realistic simulation environment for the reliable turn-off of the RC-IGCT.

**Fig. 14:**
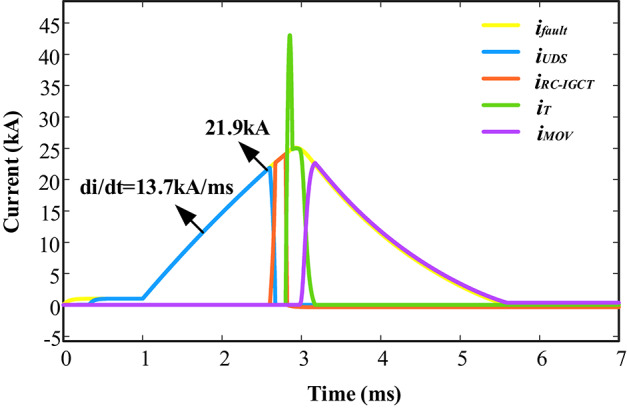
Overall waveforms of SDCCB under fault without H-SFCL.

To compare the current suppression effect of H-SFCL, the overall waveforms of the proposed SDCCB operated under faults without H-SFCL is shown in Fig. 14. It can be seen that, without H-SFCL, the short-circuit current can reach up to 21.9 kA.

**Fig. 15:**
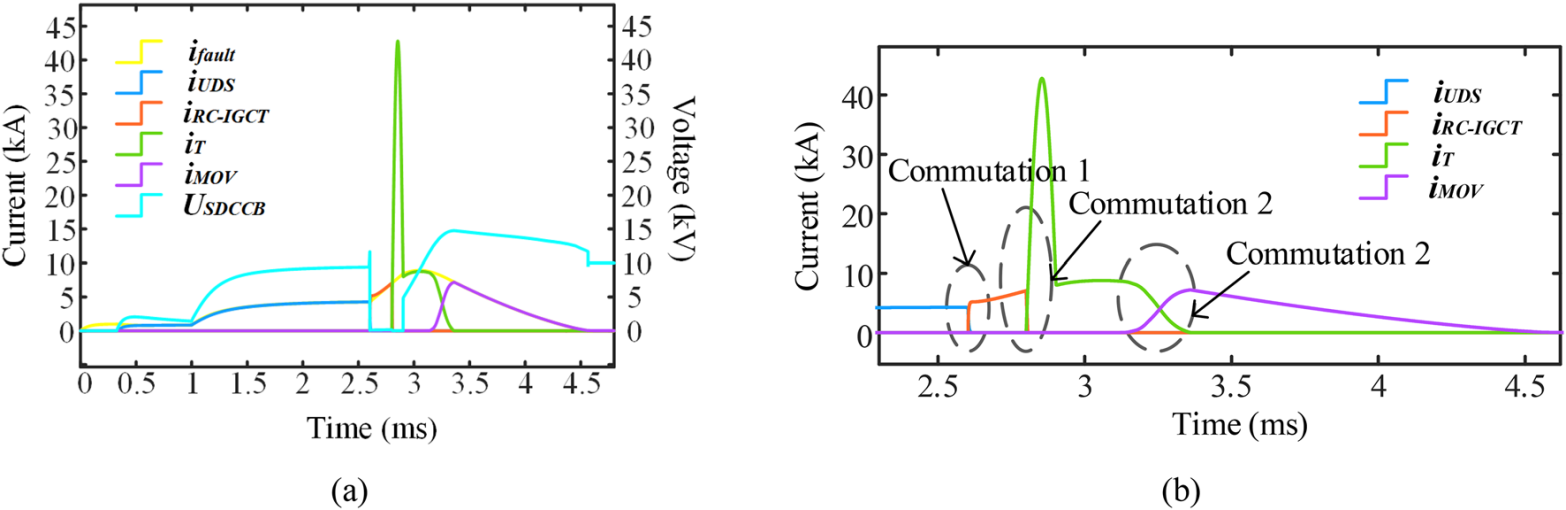
Main Process of SDCCB Operation under Fault. (**a**) overall waveform. (**b**) detailed commutation waveform.

Figure 15 (a) shows the simulated waveform of a novel superconducting hybrid DC circuit breaker based on RC-IGCT applied in a 10 kV DC test system. The entire breaking operation is completed within 3.5 ms. During normal operation of the DC system, the H-SFCL and UDS remain closed. Assuming a fault occurs at t = 1 ms, the fault current flows through the main branch. At this point, the H-SFCL begins to operate, limiting the current from 21.9 kA to within the 4.2 kA range. At t = 2.6 ms, the arc in the UDS of the main branch gradually extinguishes, and the RC-IGCT begins to conduct. The rated current transfer branch bears the fault current until the UDS of the main current branch trips to the open position. At t = 2.8 ms, the rated current transfer branch begins to lock the current, and the thyristor in the active current injection branch is triggered. Subsequently, the LC circuit begins oscillating, and system current flows through the capacitor. At t = 2.9 ms, the voltage across the SDCCB rises due to capacitor charging. When the voltage reaches the clamping voltage, the MOV begins conducting, and the system fault energy dissipates until termination. At t = 4.5 ms, when the line current drops to zero, the fault is cleared, and the circuit breaker completes its closing operation.

Figure 15 (b) shows the detailed waveform of the current interruption. Three commutation processes occur throughout the disconnection operation. First, the fault current transfers from the main branch to the rated current commutation branch. During this process, the H-SFCL limits the current from 21.9 kA to within the 4.2 kA range. The second transition involves transferring the fault current from the rated current conversion branch to the active current injection branch. The third transition transfers the fault current from the active current injection branch to the MOV branch.

The novel superconducting hybrid DC circuit breaker based on RC-IGCT proposed in this paper possesses a fault current interrupting capacity of 10 kV/21.9 kA, capable of interrupting fault currents within 3.5 ms. This fault current interrupting capability also depends on the device’s rated parameters, which will be detailed in the subsequent content.

### Performance comparison of A-IGCT, RC-IGCT, and IGBT for circuit breaker applications

This section focuses on comparing the breaking performance of three types of solid-state switches, A-IGCT, RC-IGCT, and IGBT, in the proposed novel superconducting hybrid DC circuit breaker. Under the premise of an unchanged topology, different solid-state switches require corresponding parameter configurations for their active current injection branches. The specific settings are based on a safe engineering margin design and are detailed in Table 2.

**Table 2:** Active Current Branch Parameter Settings.

	Rated Voltage / Rated Current	C_1_	U_C1_	L
IGBT	4.5 kV/2 kA	0.4 mF	5 kV	7 µH
A-IGCT	4.5 kV/4 kA	0.3 mF	4 kV	4 µH
RC-IGCT	5.5 kV/1.8kA	0.3 mF	5 kV	4 µH

**Fig. 16:**
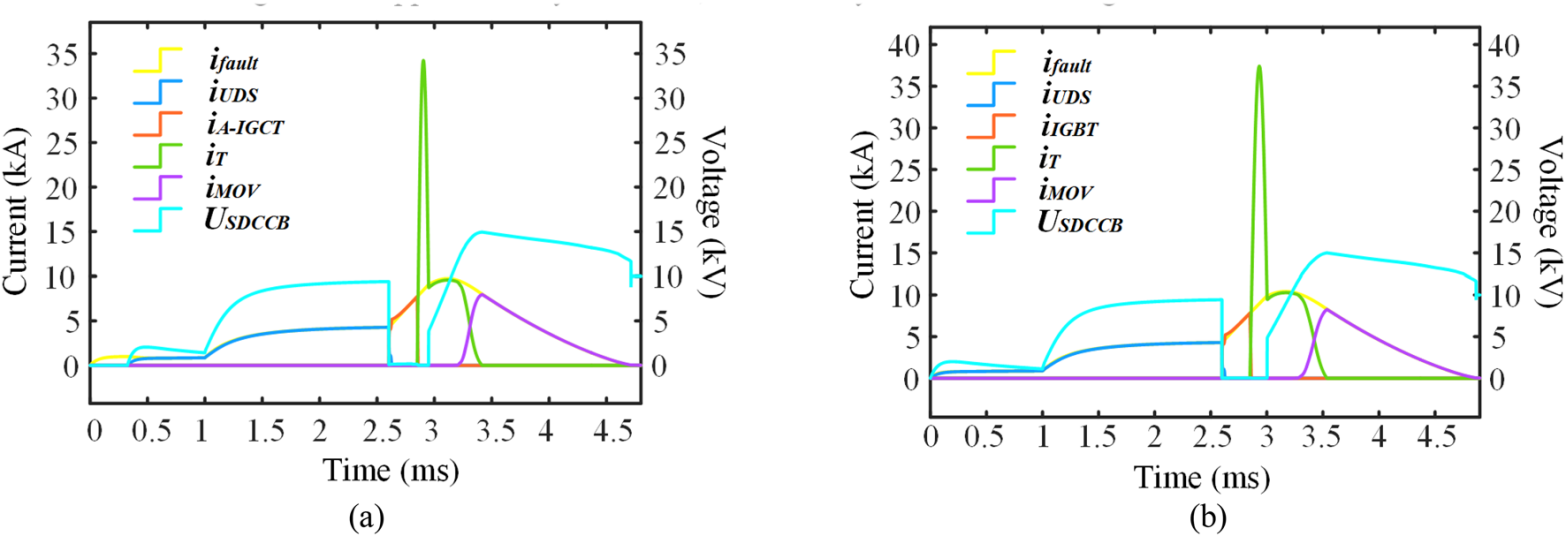
Current interruption waveforms of circuit breakers. (**a**) Current interruption waveform of a novel superconducting hybrid DC circuit breaker based on A-IGCT; (**b**) Current interruption waveform of a novel superconducting hybrid DC circuit breaker based on IGBT.

Figure 16 shows the current breaking waveforms of the SDCCB based on A-IGCT and IGBT, respectively. Compared with the waveform of the RC-IGCT-SDCCB in Fig. 14(a), it can be observed that in the medium-voltage DC system, all three types of solid-state switches can achieve effective fault current interruption. However, due to differences in device characteristics, the breaking time and the fault current borne by the thyristor in the injection branch vary: the A-IGCT-SDCCB can interrupt the fault current within 3.7 ms, with the thyristor in the injection branch withstanding a fault current of 34.2 kA; while the IGBT-SDCCB has a breaking time of approximately 4.866 ms, with the thyristor withstanding a fault current of 37.4 kA.

To distinguish the effects of device characteristics and injection branch parameters on performance, this paper supplements two sets of controlled variable analyses: one fixes the type of solid-state switching device while varying the parameters of the active injection branch; the other fixes the parameters of the active injection branch while substituting different solid-state switching devices. The comparative analysis results are summarized in Table 3.

**Table 3:** Breaking time of the circuit breaker under controlled variable experiments.

	A-IGCT-SDCCB	RC-IGCT-SDCCB	IGBT-SDCCB
L = 4 µH, C1 = 0.3 mF and UC1 = 5 kV	3.67ms	3.5ms	3.674ms
L = 4 µH, C1 = 0.3 mF and UC1 = 4 kV	3.7ms	3.56ms	3.72ms
L = 7 µH, C1 = 0.4 mF and UC1 = 5 kV	3.86ms	3.65ms	3.866ms

The comparison results indicate that regardless of the parameter settings, the RC-IGCT-SDCCB consistently exhibits the shortest breaking time. The differences in breaking time among the proposed circuit breakers primarily stem from the inherent characteristics of the different solid-state switching devices, which fully demonstrates the superiority of the RC-IGCT in terms of switching speed. Furthermore, under the premise of keeping the device type unchanged, increasing U_C1_ increases the injection branch current while shortening the breaking time. Simultaneously increasing the inductance and capacitance values results in a longer breaking time and a reduced injection current amplitude. Therefore, the parameter settings of the active current injection branch are particularly important in circuit breaker design, and a detailed analysis will be provided in the following sections.

### Current limiting characteristics of SFCL

To coordinate with circuit breaker tripping, current limiters must operate during the capacitor discharge phase and withstand short-duration DC overcurrent surges, short-term voltage stresses, and thermal shocks. Simultaneously, current limiters require rapid response capabilities to limit short-circuit currents immediately upon fault initiation. This study simulated and analyzed the fault current response of R-SFCL, SI-SFCL, novel SFCL, and H-SFCL. Given the differing current-limiting principles of the four SFCL types, selected parameters were designed to limit current to within 70% within a 1-millisecond timeframe. Parameters for the different SFCLs are shown in Table 4.

**Table 4:** Key Parameters of Different SFCL Simulation Models.

Parameters	Value
Maximum Impedance of SI-SFCL	3 Ω
R-SFCL	R_SC_=6 Ω
H-SFCL	R_1_=3 Ω R_2_=8 Ω L_SC_=3 mH
Novel-SFCL	*R* = 5 Ω L = 0.1 H k = 0.98

**Fig. 17:**
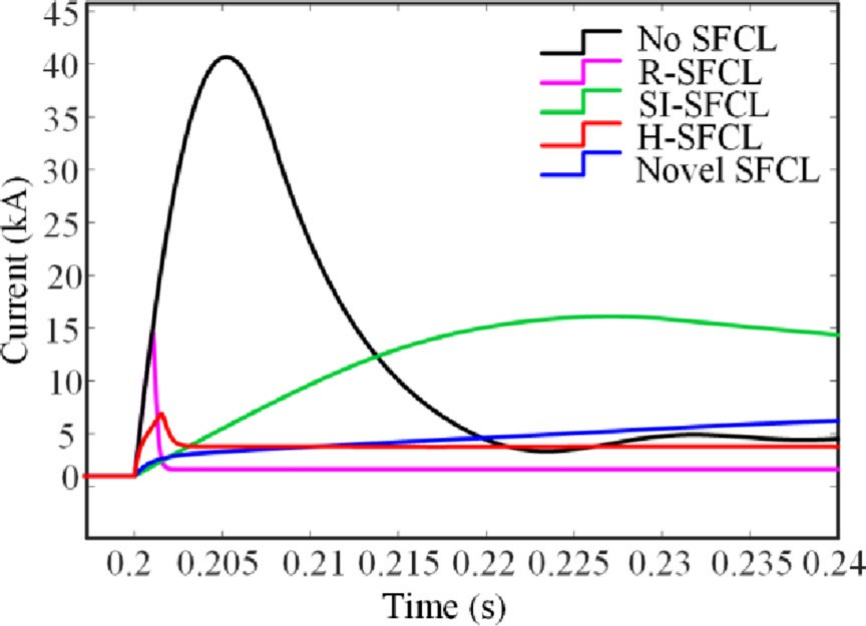
Comparison of Current Limiting Characteristics Among Four SFCL Models.

Figure 17 compares the fault current limiting effects of different SFCLs in DC systems. During the initial fault phase, rapid current rise occurs due to capacitor discharge on the DC side. At this stage, the novel SFCL and SI-SFCL demonstrate the most significant suppression of current rise rate, while the H-SFCL also exhibits excellent initial current limiting capability, outperforming the R-SFCL. As the fault progresses, the R-SFCL maintains continuous current limiting. However, the SI-SFCL and Novel SFCL exhibit limited sustained current limiting capability due to gradually saturating impedance. The H-SFCL retains some sustained current limiting capability during the mid-to-late stages of the fault. H-SFCL combines resistive and inductive characteristics, effectively suppressing current peaks and rise rates during the initial fault phase while maintaining stable current limiting performance in the middle and late phases. It also effectively controls system discharge time, preventing excessive prolongation of the discharge process that could impair system recovery speed.

### Effect of circuit parameters on current

#### Influence of current limiter parameters

To investigate the influence patterns of internal parameters in the H-SFCL, a baseline configuration of R_1_ = 3 Ω, R_2_ = 8 Ω, and L = 3 mH was established. When examining the effect of a specific variable on the system, the remaining variables were set to their corresponding baseline parameters. Given that the proposed circuit breaker action time does not exceed 5 ms, the duration was set to 5.5 ms after the short-circuit fault occurred. The following variations were tested: R_1_ at 3 Ω, 5 Ω, 7 Ω, and 9 Ω; R_2_ set to 4 Ω, 6 Ω, 8 Ω, 10 Ω, and L set to 3 mH, 5 mH, 7 mH, 9 mH. This investigation examined the effects of these three variables on the current-limiting characteristics of the DC side.

**Fig. 18:**
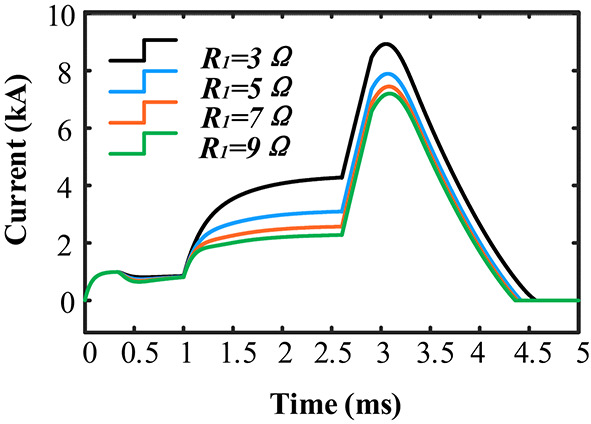
R_1_ Current Waveform at Different Parameters.

Figure 18 compares the effects of different R_1_ values. As shown, increasing R1 enhances the equivalent limiting impedance of the H-SFCL. Furthermore, increasing R_1_ mitigates the impact of DC short-circuit faults and improves the robustness of VSC-based DC systems. Therefore, R_1_ plays a crucial role in H-SFCL, primarily in system-level protection functions. However, increasing R_1_ readily leads to larger currents, higher overvoltages, and greater fluctuations in magnetic energy within the superconducting coil.

**Fig. 19:**
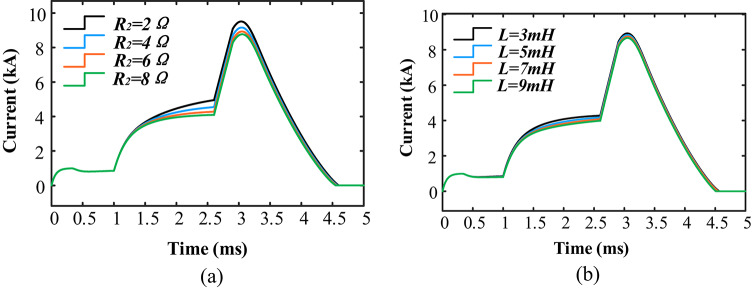
Waveforms under different parameter settings (**a**) R_2_ Current Waveform at Different Parameters (**b**) L Current Waveform at Different Parameters.

Figure 19 (a) compares the effects of different R_2_ values. Increasing R_2_ provides almost no improvement in the transient current limiting impedance of the H-SFCL. Therefore, increasing R_2_ only has a slight positive impact on compensating for DC-side voltage dips in the DC system and suppressing currents in the DC line and anti-parallel freewheeling diodes. This indicates that R_2_ is also not a decisive parameter for current limiting. Figure 19 (b) shows a comparison of effects at different L values. Increasing L_SC_ slightly improves the equivalent limiting impedance in H-SFCL. However, this limited change is insufficient to significantly enhance performance during DC short-circuit faults. Therefore, this indicates that L_SC_ is not a decisive parameter for current limiting effectiveness in VSC-based DC systems.

### Influence of stray inductance parameters in circuit breakers

The proposed RC-IGCT-based superconducting hybrid DC circuit breaker in this paper is significantly influenced by circuit parameter settings, including the inductance L_1_ in the active current injection branch, the capacitance C_1_ of the precharge capacitor, and the precharge capacitor voltage U_C1_. Simultaneously, stray inductance within the circuit also exerts a certain influence on the circuit breaker. To comprehensively evaluate the combined effects of parameters within the current injection branch, Table 5 presents the interrupting capacity of the novel SDCCB under varying inductance and capacitance conditions.

As shown in the table, the ability of SDCCB to interrupt fault currents decreases as the inductance L_1_ increases; conversely, this capability increases with higher precharge capacitance C_1_ and precharge voltage U_C1_. When the precharge capacitance and precharge voltage remain constant, reducing the inductance to increase the switching frequency can effectively enhance the SDCCB’s interrupting capability. Provided the insulation characteristics of the precharge capacitor remain satisfactory, appropriately increasing the precharge capacitance and precharge voltage can improve interrupting reliability.

**Table 5:** Breaking Capacity of SDCCB Under Different Circuit Parameters.

L_1_	i_T_	C_1_	i_T_	U_C1_	i_T_
**1 µH**	60 kA	**0.2 mF**	35 kA	**3 kV**	25.7 kA
**2 µH**	80 kA	**0.3 mF**	42.8 kA	**5 kV**	42.8 kA
**4 µH**	42 kA	**0.4 mF**	49.1 kA	**7 kV**	77 kA
**6 µH**	35 kA	**0.5 mF**	55 kA	**9 kV**	60 kA

**Fig. 20:**
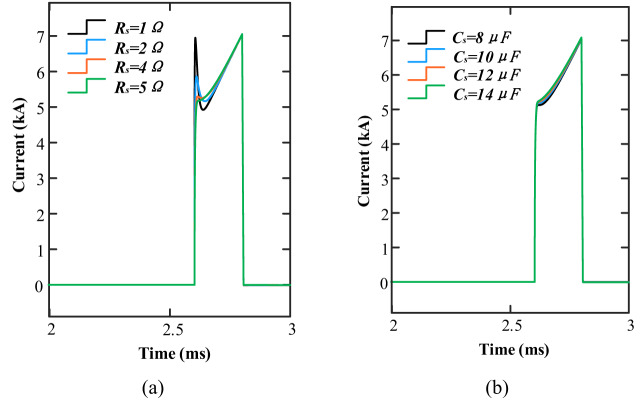
Current waveforms under different parameter settings. (**a**) Current waveforms under different R_s_ settings (**b**) Current waveforms under different C_s_ settings.

Figure 20(a) compares the effects of different Rs values. As shown, larger Rs results in smaller current spikes and smoother current waveforms. During commutation, Rs dissipates part of the electromagnetic energy in the circuit and limits the rapid discharge rate of the capacitor. Essentially, it serves as a design parameter balancing overvoltage suppression with energy dissipation. Figure 20(b) compares the effects of different Cs values. As shown in the figure, variations in Cs exert a minor influence on the current waveform. This indicates that, under this specific topology and parameter configuration, the dynamic characteristics of the main circuit are primarily governed by the main capacitance C_1_ and loop inductance L_1_, with the capacitive effects of the buffer branch being significantly masked. Consequently, the primary function of Cs likely lies not in shaping the current waveform, but rather in forming an RC buffer circuit with Rs to absorb voltage spikes during switching and suppress electromagnetic interference.

### Cost analysis

Currently proposed hybrid DC circuit breakers still require energy dissipation when fault currents are interrupted by HDCCBs. Therefore, MOVs are employed to address this excess energy. To ensure successful dissipation of system fault energy, MOV parameter design involves a trade-off between cost and performance, leaving no room for optimization. Consequently, this paper proposes the H-SFCL to alleviate the stress on MOVs. Following the current-limiting module, the current flowing through MOVs is significantly reduced in both magnitude and duration.

**Fig. 21:**
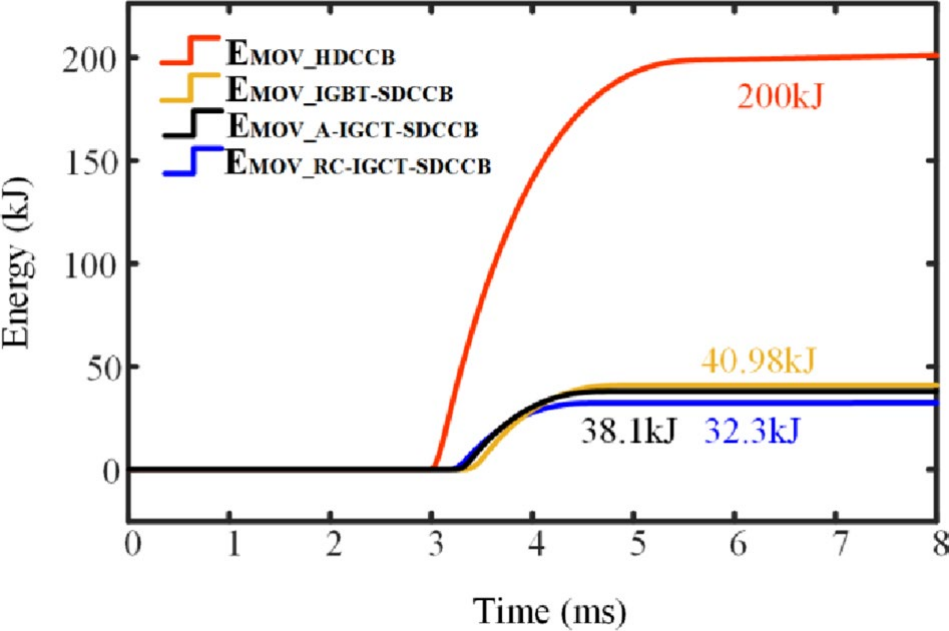
Comparison of energy absorption processes.

As shown in Fig. 21, E_MOV_HDCCB_ represents the MOV energy absorption value of the proposed superconducting hybrid DC circuit breaker without the H-SFCL current-limiting module. E_MOV_IGBT-SDCCB_ denotes the MOV energy absorption value of the IGBT-based SDCCB. E_MOV_A-IGCT-SDCCB_ indicates the MOV energy absorption value of the A-IGCT-based SDCCB. E_MOV_RC-IGCT-SDCCB_ represents the MOV energy absorption value for the RC-IGCT-based SDCCB. Compared to the HDCCB, the proposed circuit breaker reduces the energy absorbed by the MOV during switching by 167.7 kJ, representing an 83.85% decrease in MOV energy absorption. Consequently, the H-SFCL reduces the required MOV energy level, thereby lowering both the cost and size of the MOV.

At equivalent voltage and current ratings, refrigeration systems for various SFCLs show no significant differences. The demand for superconducting tapes serves as one of the cost metrics. The amount of superconducting tape required is calculated based on the overcurrent level it must withstand, with different SFCLs requiring varying quantities. Table 6 lists the normalized superconducting tape consumption for various SFCL types, using SI-SFCL as the baseline. The values for other types represent multiples relative to this baseline.

The superconducting tapes selected are second-generation high-temperature superconducting tapes with a critical current of 500 A, priced at ¥300 per meter^32^. Among these, R-SFCL accounts for the largest volume and highest cost. On one hand, the superconducting coils must withstand peak overcurrent surges exceeding 20 kA during short-circuit faults, necessitating multiple tapes connected in parallel to distribute the current and prevent tape burnout. On the other hand, to meet system current-limiting requirements, the demagnetization resistance can only be increased by extending the ribbon length. The superconducting coil in the saturated core current limiter functions solely as an excitation coil; it does not demagnetize during current limiting and does not directly bear the impact of short-circuit currents. Consequently, the saturated core current limiter uses the least amount of ribbon. The inductive component of the hybrid current limiter can be wound with conventional conductors to reduce costs. The superconducting coil employs a non-inductive structure, utilizing only its resistive component. Due to series inductive current limiting and parallel resistive shunting, the superconducting coil in hybrid current limiters experiences approximately 50% less overcurrent during short-circuit faults compared to resistive-type limiters. Consequently, the number of parallel strips can be reduced, thereby saving material consumption.

**Table 6:** The comparison of usage of superconducting tapes.

	R-SFCL	H-SFCL	SI-SFCL
Superconducting tape consumption	5.2	1	2.4

Solid-state switching devices account for the major portion of the development cost of circuit breakers; therefore, the impact of parameter adjustments in the active current injection branch on the total cost is not considered in this paper. To ensure the rationality of the cost comparison, this study selects solutions with similar breaking technologies and identical device ratings for comparison, with the relevant data presented in Table 7. It should be noted that prices may vary due to factors such as exchange rates and taxes. When comparing the costs of hybrid DC circuit breakers based on IGBTs and IGCTs, the difference in the number of components must also be taken into account. As shown in Table 6, the cost of the solution using IGCTs as the main switching devices is lower than that using IGBTs. Specifically, the proposed circuit breaker utilizing ABB’s RC-IGCT achieves a cost reduction of 37.4% compared to the A-IGCT-based solution in^33^.

**Table 7:** Performance comparison of different hybrid DC breaking schemes.

	Ref^33^	Ref^14^	Proposed topology
Breaking Capability	12 kV/15 kA	10 kV/20 kA	10 kV/21.9 kA
IGBTs	ABB 5SNA 2000K452300 ¥ 39,935 × 6	–	–
IGCTs	–	CRRC CAC4000- 45 ¥ 23,850 × 3	ABB 5SHX19L6020 ¥ 22,380 × 2
Total Cost	¥ 239,610	¥ 71,550	¥ 44,760

## Conclusions

This paper presents the design and simulation of a novel RC-IGCT-based superconducting hybrid DC circuit breaker. The breaker incorporates a H-SFCL, which successfully limits a 21.9 kA fault current to below 4.2 kA. The paper details the breaker’s topology and operating principles, establishes a simulation model, and validates its performance within a 10 kV VSC-MVDC test system. Since UDS generates arcs during current interruption, the TP KEMA arc model replaces the traditional Mayr arc model in the simulation to accurately represent arc dynamics. This paper also compares the performance of three power electronic switches in this circuit breaker, including A-IGCT, RC-IGCT, and IGBT. Among them, the RC-IGCT-SDCCB demonstrates the best performance, successfully interrupting a 21.9 kA fault current within 3.5 ms. Furthermore, the current commutation process during fault interruption is simulated under different circuit parameters. The optimized parameters for the H-SFCL are as follows: R_1_ = 3 Ω, R_2_ = 8 Ω, L_SC_ = 3 mH; the injection branch inductance L = 4 µH. Considering capacitor cost, the pre-charge capacitance is set to C_1_ = 0.3 mF with U_C1_ = 5 kV; the snubber circuit components are Rs = 5 Ω and Cs = 10 µF. In summary, this study provides a comprehensive modeling foundation, topological solution, and parameter design basis for developing high-breaking-capacity, cost-effective DC circuit breakers.

## Data Availability

The data that support the findings of this study are included within the article.
